# LINC01225 promotes occurrence and metastasis of hepatocellular carcinoma in an epidermal growth factor receptor-dependent pathway

**DOI:** 10.1038/cddis.2016.26

**Published:** 2016-03-03

**Authors:** X Wang, W Zhang, J Tang, R Huang, J Li, D Xu, Y Xie, R Jiang, L Deng, X Zhang, Y Chai, X Qin, B Sun

**Affiliations:** 1 Liver Transplantation Center of the First Affiliated Hospital and Collaborative Innovation Center For Cancer Personalized Medicine, Nanjing Medical University, Nanjing, Jiangsu Province, P.R. China; 2 Department of General Surgery, The Affiliated Jiangning Hospital of Nanjing Medical University, Nanjing, Jiangsu Province, P.R. China; 3 Department of Rheumatology, The Affiliated Hospital of Weifang Medical University, Weifang, Shandong Province, P.R. China; 4 The Affiliated Changzhou NO.2 People's Hospital of Nanjing Medical University, Changzhou, Jiangsu Province, P.R. China

## Abstract

The long noncoding RNAs (lncRNAs) have long been clarified to participate in hepatocellular carcinoma (HCC) as a biomarker. We carried out the present study in order to identify HCC-related lncRNAs and elucidate the functional roles in the development and progression of HCC. Our previous study has provided that *LINC01225* may be an HCC-related gene. Here, we verified that *LINC01225* was upregulated in HCC. Knockdown of *LINC01225* resulted in inhibited cell proliferation and invasion with activated apoptosis and cell cycle arrest *in vitro*. Overexpression of *LINC01225* in *LINC01225* knockdown cells presented that attenuated cell proliferation and invasion were restored and enhanced. Subcutaneous and tail vein/intraperitoneal injection xenotransplantation model *in vivo* validated reduced tumor progression and metastasis. Investigation of mechanism found that *LINC01225* could bind to epidermal growth factor receptor (EGFR) and increase the protein level of EGFR, and subsequently fine tune the EGFR/Ras/Raf-1/MEK/MAPK signaling pathway. Analysis with clinicopathological information suggested a high expression of *LINC01225* is positively associated with poor prognosis. We also proved *that LINC01225* was stably expressed in serum and can act as a novel biomarker in predicting the diagnosis of HCC. As a conclusion, *LINC01225* plays a crucial role in HCC and can act as a biomarker for the diagnosis and prognosis of HCC.

Hepatocellular carcinoma (HCC) accounts for 70–90% of primary liver cancer, which has emerged as the fifth most common cancer and the second leading cause of cancer-related death worldwide.^1, 2^ Tumor biomarkers may serve as an early indicator of cancer.^3, 4, 5^ However, few biomarkers have been identified associated with the genesis, growth and metastasis of HCC. It is of paramount importance to elucidate the relationship between clinical symptoms and molecular changes in HCC in order to identify new strategies for diagnosis and treatment and improve prognosis.

Long noncoding RNAs (lncRNAs) range from 200 nt up to ~100 kb in length. During the past decade, several studies have implicated that lncRNAs play pivotal roles in physiological and pathological processes.^6^ Deregulated lncRNAs were identified in HCC; several of which were confirmed as biomarkers for predicting survival and metastasis of HCC.^7, 8, 9, 10^ However, the function and clinical significance of most lncRNAs in the development of HCC remain largely unknown. Transcriptomic sequencing and bioinformatics analyses are widely used to detect lncRNAs within the human genome and identify putative candidate genes involved in carcinogenesis. Based on such methods, we found that an lncRNA, *LINC01225*, was deregulated in HCC. Investigations of its function and mechanism in combination with clinical information analysis consistently suggested that *LINC01225* is a potential diagnostic biomarker and therapeutic target for HCC.

## Results

### High expression of LINC01225 in HCC

**Figure 1:**
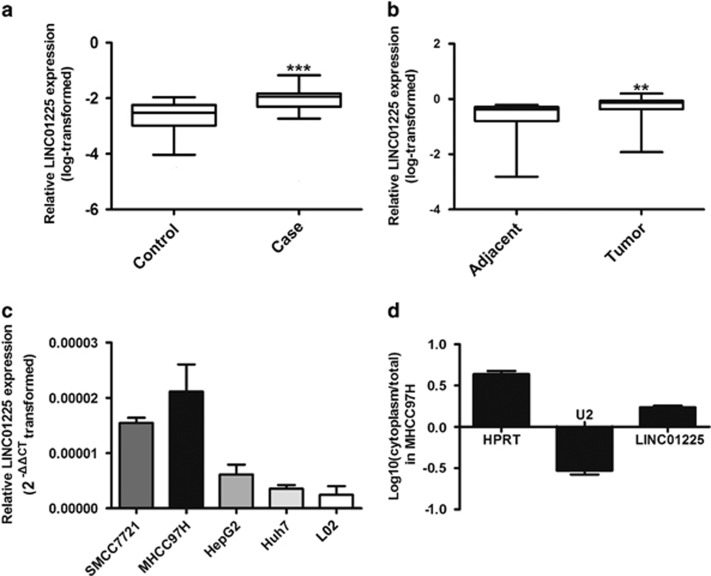
Aberrant upregulation of *LINC01225* in HCC; subcellular location. (**a**) Increased level of *LINC01225* in HCC serum (*n*=66) was detected in comparison with serum from healthy controls (*n*=70). The relative expression of *LINC01225 versus* Has-5s, calculated by 2^−△△CT^, was log transformed and presented as box plots. Box plot explanation: upper horizontal line of box, 75th percentile; lower horizontal line of box, 25th percentile; horizontal bar within box, median; upper horizontal bar outside box, 95th percentile; lower horizontal bar outside box, 5th percentile. (**b**) Increased level of *LINC01225* was detected in HCC tissues compared with the corresponding adjacent tissues (*n*=180). The relative expression of *LINC01225 versus* Has-5 s, calculated by 2^−△△CT^, was log transformed and presented as box plots. Box plot explanation: upper horizontal line of box, 75th percentile; lower horizontal line of box, 25th percentile; horizontal bar within box, median; upper horizontal bar outside box, 95th percentile; lower horizontal bar outside box, 5th percentile. (**c**) Level of *LINC01225* was detected in HCC cell lines and L02 cells. Data were 2^△△CT^ transformed and presented as mean±S.E.M. (**d**) Subcellular localization indicated that the transcript for *LINC01225* was located primarily in the cytoplasm of MHCC97H cells, according to quantitative real-time PCR amplification with separated cytoplasmic and nuclear RNA. *U2* was used as the control for cytonuclear expression and *HPRT* for cytoplasmic expression. Data were log transformed and presented as mean±S.E.M. Human Has-5 s was used as control. (**P<0.01, ***P<0.001)

In our previous study, microarray detection and bioinformatics showed that expression of *LINC01225* (mentioned as LOC149086) was increased in serum from patients with HCC.^11^ In consideration of the detection bias attributed to the limited samples provided, a large hospital-based case–control study was performed. As the result of real-time PCR showed, the *LINC01225* expression level in serum from patients with HCC was elevated in comparison with serum from healthy controls, which verified the result of microarray analysis (Figure 1a). This suggested that *LINC01225* was abnormally expressed in HCC. To study the relationship between *LINC01225* and HCC, we detected expression of *LINC01225* in 180 pairs of HCC tumors compared with the corresponding adjacent tissues by real-time PCR, which suggested that *LINC01225* transcript levels were higher in tumor tissues compared with non-tumor tissues (Figure 1b). Moreover, expression of *LINC01225* was detected in several HCC cell lines, including SMCC7721, MHCC97H, HepG2 and Huh7 and normal liver cell line, L02 (Figure 1c). The result showed that *LINC01225* is unregulated in some HCC cell lines. In addition, we detected the location of *LINC01225* transcript by real-time PCR amplified with separated nuclear and cytoplasm RNA and found *LINC01225* was located primarily in the cytoplasm of MHCC97H cells (Figure 1d).

The analysis for clinicopathological information of 180 HCC patients showed that high *LINC01225* expression was significantly associated with tumor size, tumor differentiation grade, tumor capsular integrity, tumor TNM stage and metastasis (Table 1). Meanwhile, the analysis with HCC patients' plasma also suggested that high *LINC01225* expression was significantly associated with tumor size, tumor differentiation grade, tumor capsular integrity, tumor TNM stage and metastasis (Table 2).

### LINC01225 promotes proliferation and invasion of HCC cells *in vitro*

**Figure 2:**
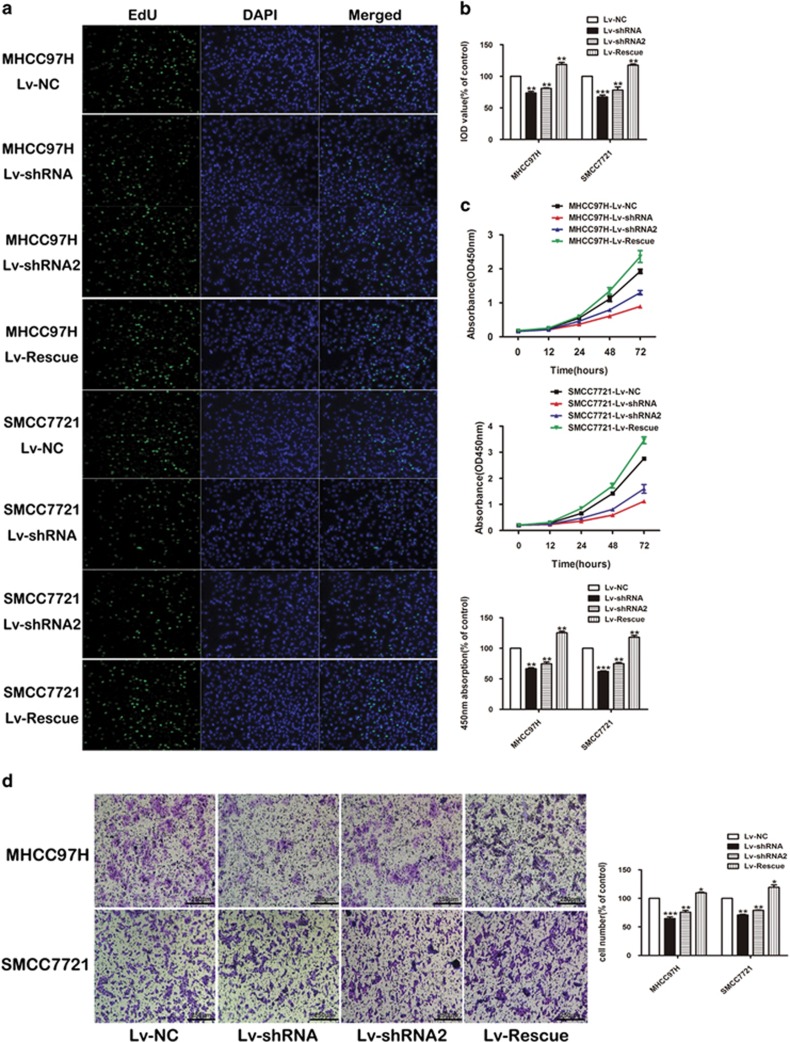
*LINC01225* promoted cell proliferation and invasion *in vitro*. (**a**) Edu assay confirmed the functional role of *LINC01225* in cell proliferation (× 200). (**b**) The results of Edu assay. Decreased level of *LINC01225* reduced the proliferation of SMCC7721 and MHCC97H after 24 h, compared with the control cells; overexpression of *LINC01225* in *LINC01225* knockdown cells restored and enhanced cell proliferation after 24 h. The integral optical density (IOD) values of cells treated with control plasmids were normalized to 100%. (**c**) Upper panels: CCK8 assay showed that *LINC01225* stable knockdown inhibited growth of SMCC7721 and MHCC97H cells; cell growth was restored and enhanced in *LINC01225* knockdown cells treated with Lv-Rescue plasmid. Lower panel: Absorbance at 450 nm was presented as the mean±S.E.M. Absorbance at 450 nm of cells treated with control plasmids was normalized to 100%. Data were collected and provided at 24 h after cultivation. (**d**) Left panels: Morphology of invasive SMCC7721 and MHCC97H cells after stable transfection with negative control, *LINC01225* shRNA or Lv-Rescue plasmid (× 400). Right panel: The number of cells treated with control plasmid was normalized to 100%, and data are presented as the mean±S.E.M., based on at least three independent experiments (*P<0.05, **P<0.01, ***P<0.01)

In order to detect the function of *LINC01225* in regulating cell biological behavior, SMCC7721 and MHCC97H were selected as high-expression *LINC01225* cells. shRNA1 and shRNA2 plasmid constructed with lentivirus plasmid, named Lv-shRNA and Lv-shRNA2 respectively, was used to knockdown the expression of *LINC01225* and further applied to detect the biological significance of *LINC01225* in tumor growth and metastasis (Supplementary Figure S1A and B). The results of EdU assay (Figure 2a) and CCK8 assay (Figure 2b) indicated that the reduced expression of *LINC01225* significantly inhibited the proliferation of SMCC7721 and MHCC97H cells. Overexpression of *LINC01225* in *LINC01225* knockdown cells presented that attenuated cell proliferation was restored and enhanced.

The Transwell assay was conducted to determine the functional role of *LINC01225* in cell invasion. Cell migration was significantly reduced in SMCC7721 and MHCC97H cells treated with shRNA in contrast to control cells; cell migration was restored and enhanced in cells treated with Lv-Rescue plasmid (Figure 2c).

**Figure 3:**
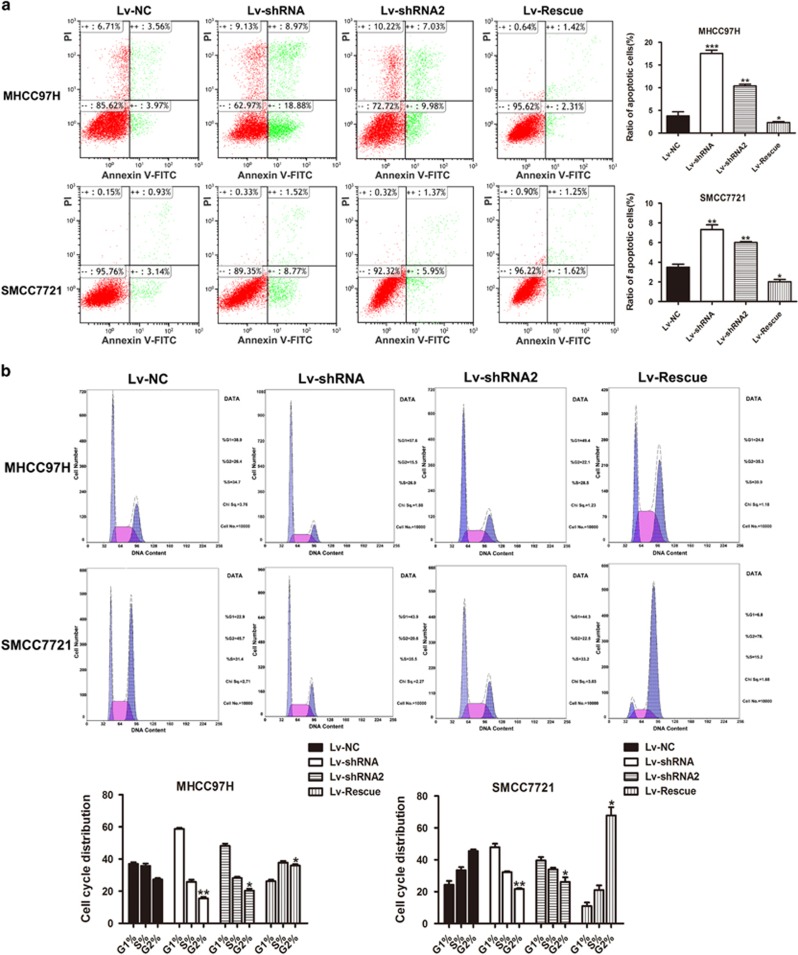
Knockdown of *LINC01225* resulted in activated apoptosis and cell cycle arrest. (**a**) Left panels: Forty-eight hours after treatment with negative control, *LINC01225* shRNA or Lv-Rescue plasmid, cells were stained and analyzed by flow cytometry. LR, early apoptotic cells; UR, terminal apoptotic cells. Right panels: Ratio of early apoptotic cells was collected and presented in the column chart. (**b**) Upper panels: At 48 h after treatment with negative control, *LINC01225* shRNA or Lv-Rescue plasmid, cell cycle was analyzed by flow cytometry. Lower panels: The bar chart represents the percentage of cells in the G1, S or G2 phase. All experiments were performed in triplicate and presented as the mean±S.E.M. (*P<0.05, **P<0.01, ***P<0.001)

FACS technology was applied to determine the influence of *LINC01225* on apoptosis. The results suggested that cells with decreased expression of *LINC01225* had a higher level of apoptosis (Figure 3a). The detection of the role of *LINC01225* in the regulation of cell cycle showed that treatment with shRNA resulted in observable cell cycle arrest (Figure 3b). Overexpression of *LINC01225* in *LINC01225* knockdown cells have restored the cell ability with low level of apoptosis and reduced cell cycle arrest (Figures 3a and b). Accordingly, we speculated that *LINC01225* could promote cell proliferation and invasion.

### LINC01225 acted as a promoter of tumor growth and metastasis *in vivo*

**Figure 4:**
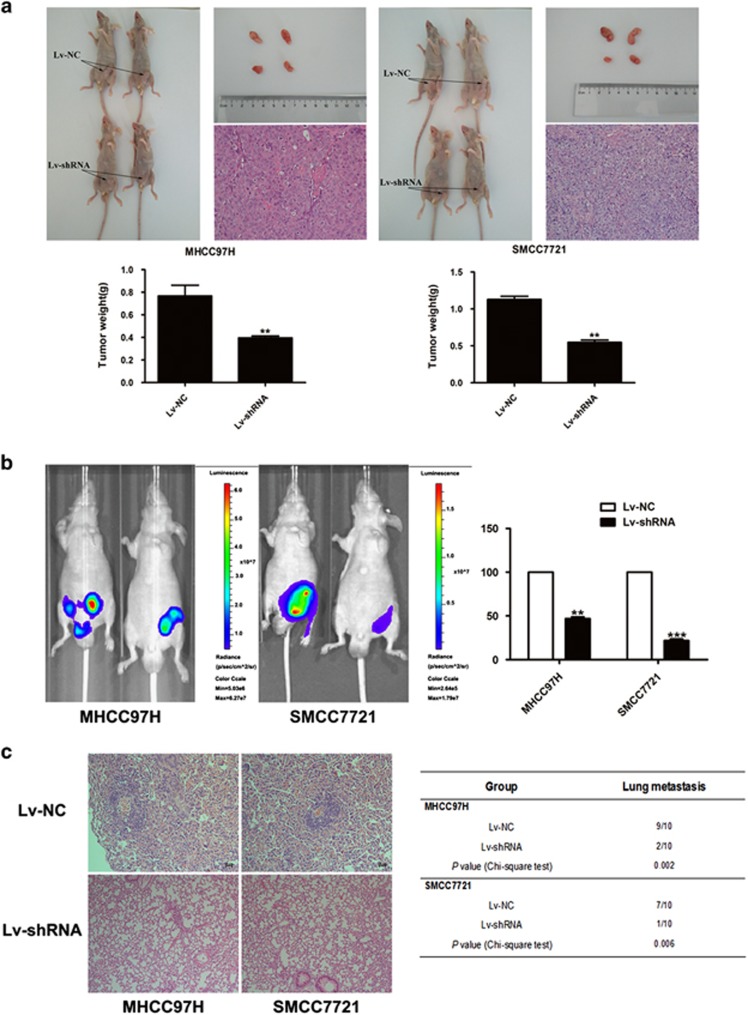
*LINC01225* downregulation inhibited tumor growth and metastasis due to *LINC01225* knockdown *in vivo*. (**a**) Upper panels: The right groin of BALB/C nude mice was subcutaneously transplanted with SMCC7721 or MHCC97H cells stably transfected with *LINC01225* shRNA or control plasmids (*n*=5). As indicated by arrows, 4 weeks after implantation, *LINC01225* depletion attenuated tumor growth in nude mice. Lower panels: The weight of each tumor was measured. Data are presented as the mean±S.E.M. H&E staining of the tumors is shown (× 400). (**b**) Left panels: Tumor model of intraperitoneal transplantation with *LINC01225* stably knocked down cells or control cells in BALB/C nude mice was constructed to detect abdominal metastases. Fluorescence intensity was measured using the IVIS Lumina II system. Mice injected with SMCC7721 and MHCC97H cells stably knocked down with *LINC01225* suppressed metastasis of tumor cells compared with controls. Right panel: Fluorescence intensity in control cells was normalized to 100% data were presented as the mean±S.E.M. (**c**) Left panels: Representative figures of tail vein xenograft model indicated lung colonization, which was formed in SMCC7721 and MHCC97H cells (*n*=10). Right panel: The number of mice with pulmonary metastastic foci was calculated in each group, as presented in the table. Lung colonization was formed in 7/10 SMCC7721 control group mice and 9/10 MHCC97H control group mice; 1/10 of the LINC01225 stably knocked down SMCC7721 group and 2/10 of the *LINC01225* stably knocked down MHCC97H group also had lung colonization. The *χ*^2^ test was used to analyze the difference. H&E staining of the lung tissues is shown. (× 400). All experiments were performed in triplicate and presented as the mean±S.E.M. (**P<0.01, ***P<0.001)

A xenotransplantation model was used to determine the role of *LINC01225* in tumorigenesis. Compared with the control group, tumors in mice injected with SMCC7721 and MHCC97H cells transfected with Lv-shRNA were significantly smaller (Figure 4a). Hematoxylin and eosin (H&E) staining of the tumors is shown in Figure 4a. Detection of abdominal metastasis in a nude mouse model transplanted intraperitoneally with *LINC01225* stably knocked down cells or control cells indicated that decreased expression of *LINC01225* was significantly related to reduced invasion and abdominal metastases of SMCC7721 and MHCC97H cells in accordance with photon flux detection (Figure 4b). Lung metastases formed in 7/10 SMCC7721 control group mice and 9/10 MHCC97H control group mice, and 1/10 SMCC7721-Lv-shRNA group mice and 2/10 MHCC97H-Lv-shRNA mice (Figure 4c). These results were verified by histological examination.

### LINC01225 promotes HCC growth and invasion via EGFR/MAPK pathway

Microarray was performed with *LINC01225* stable knockdown cells and mock empty plasmid of pll3.7-treated cells. As shown in Supplementary Figure S1C, 4/0.25 was set as the cutoff values to select candidate aberrant expression genes for gene set enrichment analysis. As indicated by gene-annotation enrichment analysis, the mitogen-activated protein kinase (MAPK) signaling pathway had a high correlation with downregulation of *LINC01225* (Supplementary Figure S1D).

**Figure 5:**
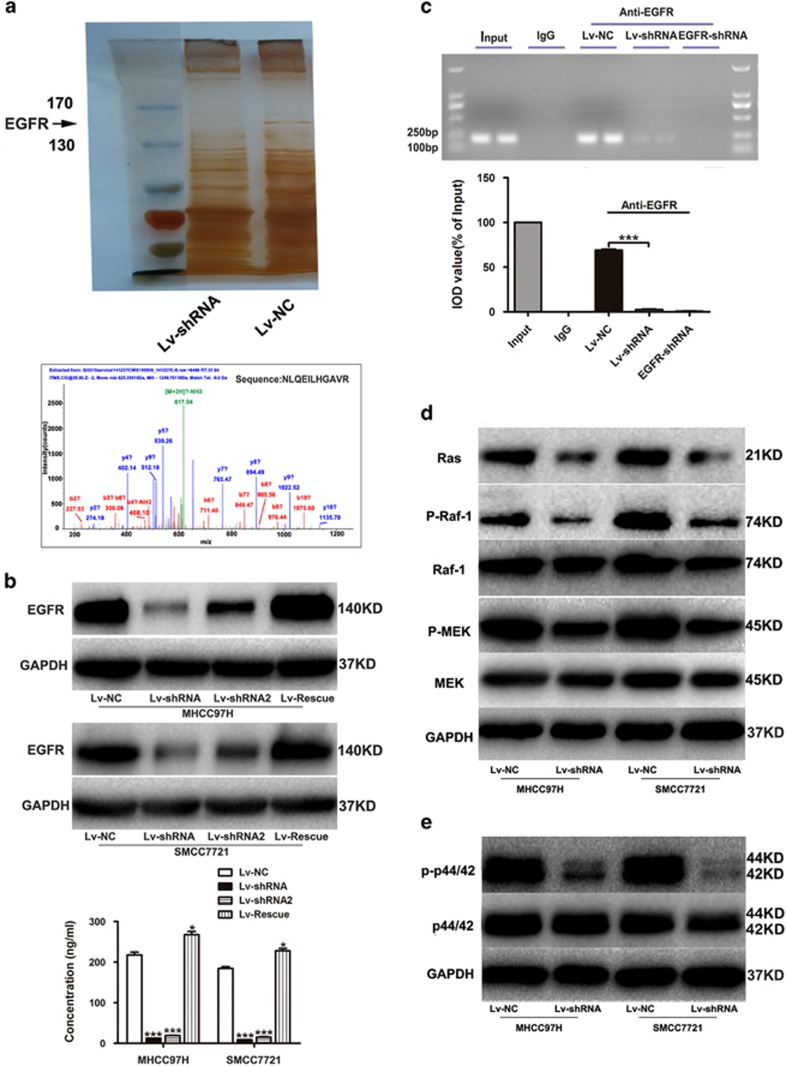
*LINC01225* promotes HCC growth and invasion via the EGFR/MAPK pathway. (**a**) Upper panel: Polyacrylamide gel stained with rapid silver staining showed that EGFR was reduced in *LINC01225* knocked down MHCC97H cells. Lower panel: MS analysis was performed to discover abnormally expressed protein due to *LINC01225* depletion. Trypsin digestion fragment detected by MS was presented in the panel. Other fragments detected were VLGSGAFGTVYK, YSFGATcVK, cNLLEGEPR, EISDGDVIISGNK, ELIIEFSK, ELVEPLTPSGEAPNQALLR, ESDcLVcR, GDSFTHTPPLDPQELDILK and RPAGSVQNPVYHNQPLNPAPSR. b and y stands for N-terminal and C-terminal collision-induced dissociation fragment ions. (**b**) Upper panel: the EGFR protein expression level was detected by western blotting. Lower panel: ELISA was used to detect the protein of EGFR in cell lysate. Cells were treated with control plasmid, Lv-shRNA, Lv-shRNA2 and Lv-Rescue plasmid. (**c**) Upper panel: RIP was performed using an antibody against EGFR on extracts from MHCC97H cells with IgG as a negative control and MHCC97H cells treated with EGFR-shRNA as an additional control. Enrichment of *LINC01225* was normalized to the input. Purified RNA was used for real-time PCR analysis. Lower panel: Bands were detected from the RNA in the group with anti-EGFR, and attenuated bands were detected in *LINC01225* knockdown cells. (**d**) Expression of proteins participating in the EGFR/MAPK pathway detected by western blotting. Protein level of Ras, activated Raf-1 and MEK was reduced by *LINC01225* depletion. (**e**) p44/42 and activated p-p44/42 protein expression level was detected by western blotting. The protein level of activated p44/42 was decreased when *LINC01225* was knocked down. All experiments were performed in triplicate and presented as the mean±S.E.M. (*P<0.05, ***P<0.001)

Mass spectrometry (MS) analysis suggested that EGFR was decreased due to *LINC01225* depletion (Figure 5a), which was in accordance with the result of microarray analysis (data not shown). Thus, we selected EGFR as a target of *LINC01225*, through which it might regulate the MAPK signaling pathway. The different expression of EGFR was confirmed by both quantitative real-time PCR, western blotting and enzyme-linked immunosorbent assay (ELISA) assay (Figure 5b and Supplementary Figure S1E). We found that there was more significant change at the protein level than at the mRNA level. Additionally, the *LINC01225* transcript was located primarily in the cytoplasm of MHCC97H cells, as indicated by the results of real-time PCR amplified with separated nuclear and cytoplasm RNA (Figure 1d), which demonstrated that *LINC01225* may interact with proteins in the cytoplasm, rather than acting as a novel transcription factor. We therefore hypothesized that regulation of EGFR by *LINC01225* at the protein level is more important than at the mRNA level. To verify this assumption, we carried out RNA immunoprecipitation (RIP) (Figure 5c), which showed that *LINC01225* could bind to EGFR, thereby increase the protein level of EGFR.

Western blotting indicated that the level of Ras and activated Raf-1 was reduced, along with activated MEK (mitogen-activated protein kinase/p44/42 kinase) and MAPK, phosphorylated p44/42 (Figures 5d and e). These findings demonstrated that *LINC01225* has a modifying effect on HCC via reducing the activation of the EGFR/Ras/Raf-1/MEK/MAPK pathway.

### LINC01225 as a biomarker for HCC diagnosis and prognosis

**Figure 6:**
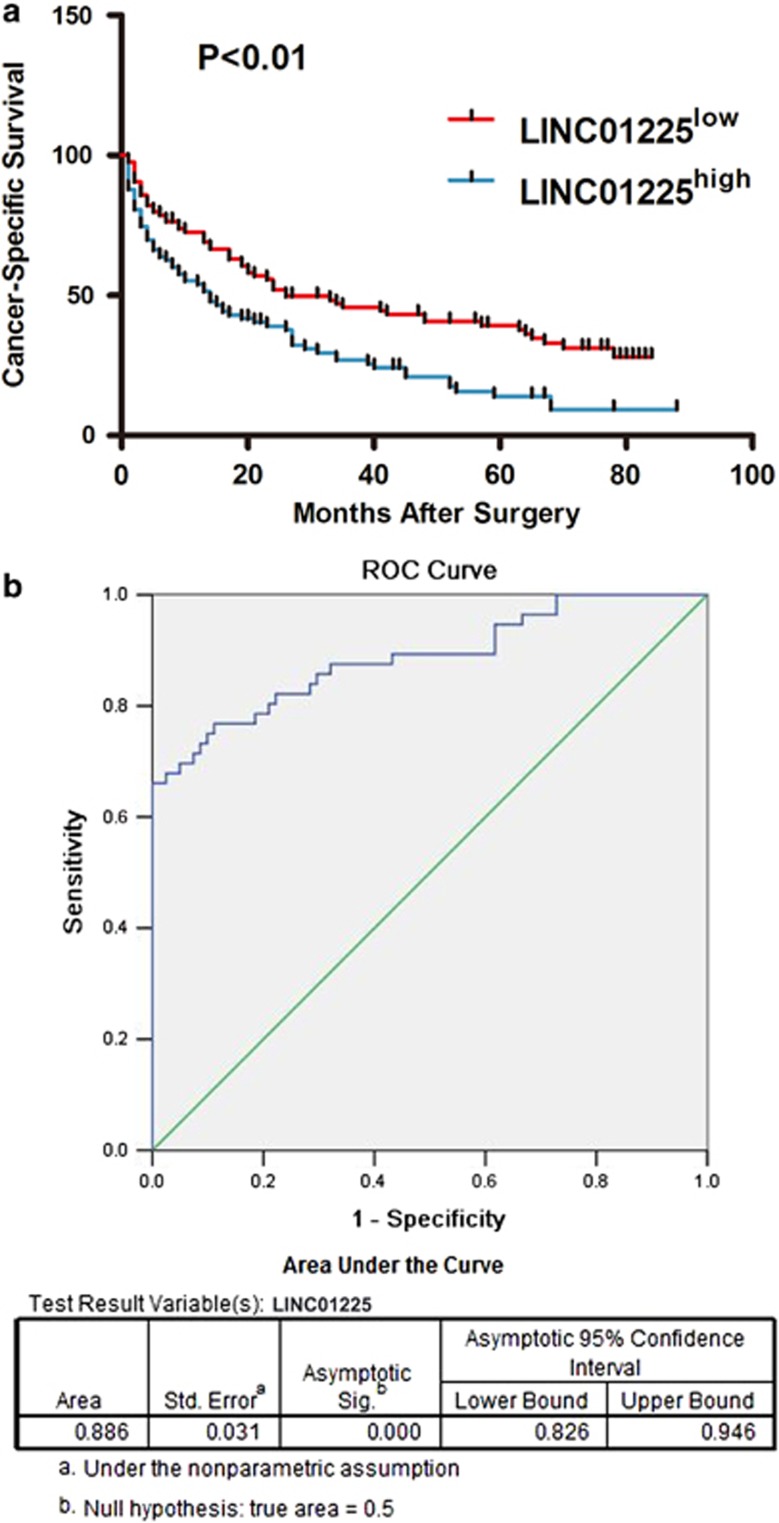
*LINC01225* as a biomarker for HCC diagnosis and prognosis. (**a**) Kaplan–Meier analysis was initiated to discover whether the expression of *LINC01225* was associated with overall survival of HCC patients. The median was used as the cutoff. (**b**) Expression of *LINC01225* was detected in patients from whom plasma was obtained preoperatively, by comparison with patients free of tumor. Receiver operating characteristic curve analysis of *LINC01225* was used to detect the diagnostic efficiency of HCC.

Previous studies have reported lncRNAs as biomarkers for diagnosis and predictors for prognosis of HCC.^7, 8, 9, 10^ We performed Kaplan–Meier analysis to identify whether expression of *LINC01225* was associated with the cancer-specific survival rate in HCC patients. With the median as the cutoff based on the expression level of *LINC01225* in HCC tissues, patients were separated into a high-level group (*n*=90) and a low-level group (*n*=90). Expression of *LINC01225* was negatively associated with cancer-specific survival (*P*<0.01) (Figure 6a). We performed risk score analysis to investigate the effectiveness of *LINC01225* for HCC prediction. Due to the limitations that serum samples corresponding to the HCC tissues could not be obtained, we therefore randomly selected several HCC serum samples (*n*=66) collected from patients before hepatectomy and control serum samples (*n*=70) were collected from people undergoing physical examination. Serum samples were frozen and thawed for five times, and expression of *LINC01225* in serum detected by qRT-PCR indicated that *LINC01225* was stably expressed in serum (Supplementary Figure S1F). LINC01225 may be an effective predictor for HCC diagnosis with a sensitivity of 0.761 and a specificity of 0.443 (Figure 6b).

## Discussion

HCC is one of the most common cancers and has a high mortality.^12^ HCC is rarely detected in the early stages because of its slow progression and early lack of symptoms. This means that patients are mostly diagnosed at an advanced stage and cannot be cured.^13, 14^ Clinical research has revealed that HCC detected at the early stages has good prognosis and high overall survival rate after treatment with transhepatic arterial chemotherapy and embolization or radiotherapy.^2, 15, 16^ On account of the significance of early detection of HCC, several methods were developed, such as traditional diagnostic imaging with ultrasound, computed tomography and diagnostic biomarkers. Tumor biomarkers are specifically expressed in some diseases or differentially expressed between patients and healthy individuals, and can be detected in body fluids or secretions. Biomarkers may be used as indicators of tumorigenesis and for surveillance of tumor progression and metastasis, as well as serving as predictors of prognosis and survival. In addition to metabolites and tumor-specific antigens, tumor-related genes have emerged as promising biomarkers with high specificity and sensitivity.^17, 18, 19^

lncRNAs regulate a wide variety of physiological and pathological processes through diverse mechanisms; however, previously, they were thought to be transcriptional noise.^20, 21^ Accumulating studies have demonstrated that lncRNAs have important functions and are associated with a wide range of diseases.^22, 23, 24, 25^ lncRNAs are involved in all aspects of gene regulation, including chromosome dosage compensation, imprinting, epigenetic regulation, nuclear and cytoplasmic trafficking, transcription, and mRNA splicing and translation.^7, 26, 27^ HCC is a complex polygenetic disease with many coding and noncoding genes involved.^28, 29^ Many studies have suggested that several lncRNAs play important roles in the development and progression of HCC, such as HOTAIR, HULC, MALAT1 and H19.^30, 31, 32, 33^ Due to the undiscovered functions of lncRNAs and limited knowledge of the underlying mechanisms, more studies are urgently needed. The present study found an HCC-related lncRNA, *LINC01225*, and elucidated its functional roles in HCC development and progression.

As described in the NCBI database, *LINC01225* is a human noncoding RNA validated by the Human Gene Nomenclature Committee. It is located at chromosome 1p35.2 with 2113 bp and has eight exons. Our previous study reported that *LINC01225* was highly expressed in serum of patients with HCC, which suggested that *LINC01225* is an HCC-related gene.^11^ In the present study, we found that *LINC01225* was highly expressed in serum and tumor tissues of patients with HCC. High expression of *LINC01225* was positively related to tumor size and metastasis, as suggested by the analysis of clinicopathological information. Our *in vitro* experiment demonstrated that the absence of *LINC01225* was responsible for reduced cell proliferation and invasiveness in HCC, and re-expression of *LINC01225* in *LINC01225* knockdown cells showed that attenuated cell proliferation and cell migration were restored and enhanced. Moreover, cell cycle arrest was obviously increased and apoptosis was markedly promoted by *LINC01225* depletion and re-expression of *LINC01225* in *LINC01225* knockdown cells has restored the cell ability with a low level of apoptosis and reduced cell cycle arrest. Our *in vivo* study of the effect of *LINC01225* showed that reduced tumor size and depressed abdominal metastasis were attributed to depletion of *LINC01225*. Lung metastatic node formation was decreased by *LINC01225* knockdown, which further suggested that *LINC01225* might act as a promoter of HCC.

EGFR plays a pivotal role in cell survival and proliferation.^34^ Consequent transformation has been observed in model systems of autocrine stimulation, overexpression and mutation of EGFR.^35, 36^ EGFR exerts its effect by activating a network of signaling elements, involving Ras, phosphoinositide 3-kinase and the signal transducer and activator of transcription family.^37, 38^ A cascade of protein kinases, with activated phosphorylation by growth factors, is well established, including tyrosine kinases, Ras, Raf-1, MEK and MAPK.^39, 40, 41^ The Ras/Raf-1/MEK/MAPK signaling pathway is of major importance in intracellular transduction of proliferative signals from activated cell membrane growth factor receptors to the nucleus, and it is involved in cell proliferation and apoptosis in several tumors including HCC.^42^ Raf-1 is a serine/threonine kinase that is activated by GTP-bound Ras, the activated type of Ras, subsequently phosphorylating downstream signal MEK, which phosphorylates and activates MAPK.^43, 44^ MAPK phosphorylates a series of cellular substrates with crucial roles in cell survival and proliferation. Whether lncRNAs modify HCC development and progression through activation of these molecules remains vague.

Previous studies have identified lncRNAs as transcriptional co-regulators, through which lncRNAs realized the regulatory roles by interacting with proteins.^45, 46, 47, 48^ Further investigation of the exact mechanism through which *LINC01225* participates in HCC is urgently needed. Microassay combined with MS had determined EGFR as a target of *LINC01225*. We also found as a result of *LINC01225* knockdown that EGFR was dramatically reduced more at the level of protein than mRNA, which suggests that *LINC01225* affects EGFR signaling primarily through regulating EGFR protein. GO and KEGG pathway analysis suggested that *LINC01225* functions as a regulator by affecting the MAPK signaling pathway.

According to clinicopathological analysis of information at follow-up, high expression of *LINC01225* is positively associated with poor prognosis. In addition, we have demonstrated *LINC01225* as a novel biomarker in predicting the diagnosis of HCC by analyzing its expression in serum.

In summary, our study shows the upregulation of *LINC01225* in HCC, and further demonstrates that *LINC01225* plays a crucial role in HCC through binding to protein and increasing the level of EGFR as a consequence, thus fine tuning the EGFR/MAPK signaling pathway. *LINC01225* may be a promising biomarker for the diagnosis and prognosis of HCC, and it can be a therapeutic target for HCC. However, more studies are warranted for further investigation of the function of *LINC01225* in the future.

## Materials and Methods

### Patients and clinical samples

Fresh HCC specimens, paired adjuvant noncancerous tissue samples and HCC serum samples were obtained from patients undergoing hepatectomy between 2008 and 2010. Serum samples from healthy persons were randomly selected at the First Affiliated Hospital of Nanjing Medical University (Nanjing, China). The study was approved by our Institutional Ethics Committee. Our research was performed in accordance with the Helsinki Declaration and government policies. Written informed consent was obtained from all participants. Peripheral blood samples were collected before hepatectomy. Specimens and corresponding clinical and pathological materials are summarized in Table 1 (tissues) and Table 2 (serum).

### Cell lines and animals

HCC cell lines SMCC7721 and MHCC97H and common human liver cell line L02 were purchased from American Type Culture Collection, and MHCC97H and Huh7 cells were obtained from the Shanghai Institute of Biochemistry and Cell Biology (Chinese Academy of Sciences, Shanghai, China). The cell lines were routinely cultured in Dulbecco's modified Eagle's medium (DMEM) (Invitrogen, Grand Island, NY, USA) with 10% heat-inactivated fetal bovine serum (FBS) (Gibco, Carlsbad, CA, USA), 2 mM l-glutamine, 100 U/ml penicillin and 100 mg/ml streptomycin, in 5% CO_2_ at 37 °C.

Male BALB/c nu/nu mice (4–8 weeks old) were purchased from the Laboratory Animal Center of Yangzhou University and maintained under specific pathogen-free conditions. All animals received humane care and all experiments were carried out according to the guidelines outlined in the Guide for the Care and Use of Laboratory Animals.

### Cell transfection

Two shRNAs of *LINC01225* sequences were designed, shRNA1: GGAUAAUUCUAAUGCCUACTTGUAGGCAUUAGAAUUAUCCTT; shRNA2: UCCAGUUGGCGAGAGUGAUTTAUCACUCUCGCCAACUGGATT. SMCC7721 and MHCC97H cells were transfected with lentivirus plasmid constructed with shRNA of *LINC01225* as previously described,^49^ and finally shRNA1 plasmid termed lentivirus-short hairpin RNA (Lv-shRNA) was selected to be used in the subsequent experiments. Lentivirus plasmid constructed with scramble sequence was used as control, termed Lv-NC. The synthesized and purified *LINC01225* gene fragment was inserted into a lentivirus vector (pll3.7), named Lv-Rescue. *LINC01225* knockdown SMCC7721 and MHCC97H cells were then transfected with the packaged recombinant lentivirus. shRNA of EGFR was designed for the target sequence: GGCTGGTTATGTCCTCATT. Lentivirus plasmid constructed with shRNA of EGFR, termed EGFR-shRNA, was used to transform MHCC97H cells as previously described^49^ (Supplementary Figure S2).

### Quantitative real-time PCR

Quantitative real-time PCR was performed to detect the expression levels of *LINC01225* and mRNAs of all related genes with SYBR Green Mastermix kit (TaKaRa, Tokyo, Japan) and analyzed in triplicate assays on the ABI Prism 7900HT (Applied Biosystems, Foster City, CA, USA) according to the direction of the reagents. Total RNAs of fresh liver samples and cells were extracted with TRIzol reagent (Invitrogen) and quantified. For RNA extraction from the cytoplast and cytoplasm, the SurePrep Nuclear or Cytoplasmic RNA Purification Kit (Life Science SOURCE; Biovision, Milpitas, CA, USA) was used. For mRNA detection, reverse transcriptase (TaKaRa) was used to reverse transcribe total RNAs (500 ng). Has-5 S was used as an internal control. Primer sequences: Has-5 S: Forward: 5′-GGAGAGGGAGCCTGAGAAACG-3′ and Reverse: 5′-TTACAGGGCCTCGAAAGAGTCC-3′ and human *LINC01225*: Forward: 5′-GTCCCTTACCTTGAGGTGCC-3′ and Reverse: 5′-CACGCCTTTGTGTTCTGGTG-3′ and human EGFR: Forward: 5′-TCCTCTGGAGGCTGAGAAAA-3′ and Reverse: 5′-GGGCTCTGGAGGAAAAGAAA-3′ were designed using Primer 3.0 software (http://www.simgene.com/Primer3).

### Western blotting and H&E staining and ELISA

Total proteins from cultured cells were extracted using radio-immunoprecipitation assay buffer plus fresh protease and phosphatase inhibitors (Beyotime, Nantong, China) and quantified with the Bradford assay (Bio-Rad Laboratories, Hercules, CA, USA). Equal amounts of protein samples (30 *μ*g) were loaded to each lane. Proteins were separated by sodium dodecyl sulfate polyacrylamide gel electrophoresis (SDS-PAGE) and then transferred to a polyvinylidene fluoride membrane. Antibodies against EGFR (Cell Signaling Technology, Danvers, MA, USA), Ras (Santa Cruz Biotechnology, Santa Cruz, CA, USA), Raf-1 (Santa Cruz Biotechnology), p-Raf-1 (Thr269) (Santa Cruz Biotechnology), MEK (Cell Signaling Technology), phosphorylated MEK (p-MEK; Cell Signaling Technology), p44/42 (ERK) (Cell Signaling Technology), p-p44/42 (Thr202/Tyr204) (Cell Signaling Technology) and human reduced glyceraldehyde-phosphate dehydrogenase (GAPDH) (Cell Signaling Technology) were used in immunoblotting. ImageJ software (NIH, Bethesda, MD, USA) was applied to quantify the integrated density of the bands. Cells suffering freezing and freeze–thaw cycles were used to extract protein for ELISA assay, which was performed with an ELISA Kit for EGFR (Uscn Life Science Inc., Wuhan, China) according to the instruction.

### Flow cytometry analysis

Apoptosis was detected with an Annexin V-FITC/PI Apoptosis Detection Kit (Vazyme Biotech, Nanjing, China) and cell cycle was determined with a Cell cycle Assay Kit (Vazyme Biotech). Cells used for apoptosis detection were treated with 0.05 mM H_2_O_2_ for 2 h for stimulation of apoptosis. Cells were finally analyzed on a FACS Calibur flow cytometer equipped with CellQuest software (BD Biosciences, New York, NY, USA).

### Cell proliferation and invasion assay

Cell proliferation was evaluated using a CCK8 kit (Vazyme Biotech) and a Cell-Light EdU Apollo567 *In Vitro* Kit (RiboBio, Guangzhou, China). For CCK8 detection, transfected cells (2 × 10^3^) were seeded into 96-well plates, cultured for 24, 48, 72 and 96 h, and then CCK8 reagent was added to each well and incubated for 2 h at 37 °C. Absorption was measured by microplate reader at 450 nm (ELX-800; Bio-Tek, Winooski, VT, USA). For 5-ethynyl-2′-deoxyuridine (EdU) assay, transfected cells (2 × 10^5^) were seeded into Glass Botttom Cell Culture Dishes (Nest Biotechnology, NJ, USA), then cells were treated according manufacture instruction, and finally cell samples were detected with a laser confocal scanning microscopy. The invasion assays were assessed using the Transwell units (Corning Costar, Tewksbury, MA, USA) percolated with Matrigel (BD Biosciences). Cells (2 × 10^4^ cells/well) were seeded at the upper compartment of the chamber in DMEM without FBS, and the lower chamber was filled with DMEM including 10% FBS as chemotaxin. After incubation for 48 h, the filters were collected, fixed with 4% paraformaldehyde and stained with 0.1% crystal violet. Non-invading tumor cells on the top of the filters were removed with cotton swabs. Cells passing through the filter were counted under a light microscopy.

### Subcutaneous xenotransplantation model

Cells (5 × 10^6^) with a stably decreased level of *LINC01225* expression in SMCC7721 and MHCC97H and control cells (Lv-NC) were subcutaneously implanted into the bilateral axillae of each BALB/C nude mice. Five weeks later, all mice were killed and the weight of each tumor was measured. Tumor tissues were integrally stripped out.

### Metastasis model

A single-cell suspension was prepared with the cells stably knocked down for *LINC01225*, or in the control group, suspended in 200 *μ*l PBS and filtered through a sterile 70-*μ*m nylon mesh filter (BD Falcon, Franklin Lakes, NJ, USA). Groups of 10 mice were separately injected with SMCC7721 and MHCC97H cells with or without knockdown of *LINC01225* via tail vein injection to develop peripheral intravascular implanted models (10 in each group). Mice were killed 5 weeks afterwards, and tumor metastasis in the lung was examined. H&E staining was used to evaluate the number of tumors in lung tissues.

In addition, a tumor model of intraperitoneal transplantation in BALB/C nude mice was constructed. Mice were injected intraperitoneally with cells treated with *LINC01225* shRNA or controls and kept in pathogen-free conditions. Abdominal metastasis was monitored weekly by the IVIS Lumina II system (Caliper Life Sciences, Hopkinton, MA, USA).

### Agarose gel electrophoresis

Agarose powder (0.5 g) was dissolved in 50 ml of 0.5% Tris-acetate-EDTA (TAE) buffer and heated to near boiling, and then 2.5 *μ*l GoldView (Beyotime) was added and mixed. Total RNA of the cell lines was extracted and reverse transcribed into cDNAs. Obtained cDNAs were mixed with loading buffer (Beyotime), and the mixture was loaded into the wells. Electrophoresis was performed with 0.5% TAE as the running buffer, at 80 V for 40 min. Data were analyzed with Image Lab software performed with an ultraviolet (UV) transilluminator.

### Microarray assay detection and bioinformatics analysis

Total RNAs extracted from MHCC97H cells including untreated cells, *LINC01225* stable knockdown cells and mock empty plasmid of PLL3.7-treated cells were amplified and used to synthesize double-stranded cDNAs. Samples prepared as required were labeled and hybridized to mRNA Human Gene Expression Microarray V4.0 (CapitalBio Corp., Beijing, China). The microarray results were used to analyze data normalization, summarization and quality control with the GeneSpring software V11.5 (Agilent, Santa Clara, CA, USA). We selected genes that were differentially expressed with a >2-fold change of threshold value, as well as Benjamini–Hochberg corrected *P*-values of <0.05. CLUSTER version 3.0 software (Stanford University School of Medicine, Stanford, CA, USA) was used to normalize and hierarchically cluster the data. Tree visualization of the data was performed with Java Treeview software (Stanford University School of Medicine, Stanford, CA, USA). Microarray data have been deposited at ArrayExpress (E-MTAB-3833).

Gene product attributes were described with three structured networks of defined terms provided with GO analysis from Gene Ontology (www.geneontology.org). The GO Term enrichment with expressed mRNA list was considered significantly different with *P*<0.05. Pathway analysis was performed on the basis of the latest KEGG (Kyoto Encyclopedia of Genes and Genomes) database for the differentially expressed mRNAs with a statistically significant *P*-value of <0.05.

### Mass spectrometry

Polyacrylamide gel was prepared in accordance with the standard protocol. Twenty microliters of each sample mixed with 10 × running buffer (CapitalBio Corp.) was loaded, and the gel was run at 120 V for 2.5 h. The gel was stained with Fast Silver Stain Kit (Beyotime). Lanes, cut into 10 pieces were respectively placed in Eppendorf tubes. NH_4_HCO_3_ (50 mM) was added and the gel was broken into pieces with the suction pipette. Then, trypsin (Promega, Fitchburg, WI, USA) was used to digest the gel solution at 37 °C overnight. Tubes were shaken and centrifuged with methyl cyanide (HPLC grade) added for 2 min at room temperature. The supernatant was transferred to the clean tubes, and dried by the vacuum concentration apparatus at 60 °C. LC-MS/MS with nano-LC combined with an Orbitrap Q Exactive mass spectrometer (Thermo Scientific, Waltham, MA, USA) was used for analysis of the peptides followed by a scan range of *m*/*z* 400–1500. Thermo Proteome Discoverer (1.4.0.288) software (http://www.thermoscientific.com/en/product/proteome-discoverer-software.html) platform was used to analyze the raw files. MS/MS spectra were retrieved against the protein database (human-refseq-20140303-71465 s.fasta, National Center of Biotechnology Information) for protein authentication.

### RNA immunoprecipitation

RIP was performed with a Magna RIP RNA-Binding Protein Immunoprecipitation Kit (Millipore, Bedford, MA, USA). An antibody for EGFR (Cell Signaling Technology) was used for RIP assays. Coprecipitated RNAs were detected using quantitative real-time PCR.

### Statistical analysis

Results of quantitative real-time PCR were expressed as mean±S.E.M. Student's *t*-test and *χ*^2^ test were used to evaluate statistical differences in clinical and demographic characteristics. Kaplan–Meier survival curves were drawn and the log-rank test was performed. Risk score analysis was done to investigate the effectiveness of the LINC01225 for prediction. Statistical analysis was performed using STATA version 9.2 (Stata Corp., College Station, TX, USA) and SPSS version 18.0 (SPSS Inc., Chicago, IL, USA) and presented with the GraphPad prism software (GraphPad Software, San Diego, CA, USA). In all cases, *P*<0.05 was considered significant.

## Figures and Tables

**Table 1:** Clinicopathological relevance analysis of LINC01225 expression in HCC patients

		**LINC01225**		
**Feather**	**All patients**	**Low expression (<median)**	**High expression (⩾median)**	***P*-value**
All cases	180	90	90	
*Age, years*				0.871
< 60	125	63	62	
⩾ 60	55	27	28	
*Gender*				0.518
Male	155	76	79	
Female	25	14	11	
*Differentiation grade*				**0.011**
Well	80	50	30	
Moderate	30	12	18	
Poorly	70	28	42	
*Tumor size (cm)*				**0.000**
⩽ 5	99	64	35	
> 5	81	26	55	
*Tumor number*				0.172
Solitary	158	82	76	
Multiple	22	8	14	
*Tumor capsular*				**0.017**
Incomplete	12	2	10	
Complete	168	88	80	
*TNM stage (I:II:III)*				**0.000**
I	85	57	28	
II	24	13	11	
III	71	20	51	
*Metastasis*				**0.000**
Yes	58	11	47	
No	122	79	43	

Total data from 180 HCC patients were analyzed. For the expression of LINC01225, the median expression level was used as the cutoff. Data were analyzed by chi-squared test. *P*-value in bold indicates statistically significant.

**Table 2:** Clinicopathological information from HCC serum samples

		**LINC01225**		
**Feather**	**All patients**	**Low expression (<median)**	**High expression (⩾median)**	***P*-value**
All cases	66	33	33	
*Age (years)*				0.218
<60	34	14	20	
⩾ 60	32	19	13	
*Gender*				0.537
Male	53	28	25	
Female	13	5	8	
Tumor size (cm)				**0.000**
< 5	30	23	7	
⩾ 5	36	10	26	
*Differentiation grade*				**0.000**
Well	8	7	1	
Moderate	33	22	11	
Poorly	25	4	21	
Tumor number				0.215
Solitary	53	29	24	
Multiple	13	4	9	
*Tumor capsular*				**0.017**
Incomplete	15	3	12	
Complete	51	30	21	
TNM stage				**0.000**
I	34	24	10	
II	16	8	8	
III	16	1	15	
*Metastasis*				**0.001**
Yes	16	2	14	
No	50	31	19	

Total data provided here were from 66 HCC patients from whom serum samples were collected. For the expression of LINC01225, median expression level was used as the cutoff. Data were analyzed by chi-squared test. *P*-value in bold indicates statistically significant.

## Abbreviations

HCC: hepatocellular carcinoma
lncRNA: long noncoding RNA
EGFR: epidermal growth factor receptor
MEK: mitogen-activated protein kinase/p44/42 kinase
ERK: extracellular signal-regulated kinase
MAPK: mitogen-activated protein kinase
RIP: RNA immunoprecipitation
shRNAs: short hairpin RNAs
Lv-shRNA: lentivirus-short hairpin RNA
GAPDH: glyceraldehyde-phosphate dehydrogenase
ELISA: enzyme-linked immunosorbent assay
IOD: integral optical density
