# Supplementary material for: LINC01225 promotes occurrence and metastasis of hepatocellular carcinoma in an epidermal growth factor receptor-dependent pathway
Source: Cell Death Dis. 2016 Mar 3;7(3):e2130–. doi: 10.1038/cddis.2016.26 (PMC4823934; doi:10.1038/cddis.2016.26)
Supplement: Supplementary Figure Legends [file cddis201626x1.doc]

**Supplementary figure legends**

**Supplementary Figure S1. A** mRNA level detected in MHCC97H cells treated with control plasmid, Lv*-*shRNA, Lv-shRNA2 and Lv-Rescue plasmid. Data were presented as the mean±S.E.M. **B** mRNA level detected in SMCC7721 cells treated with control plasmid, Lv*-*shRNA, Lv-shRNA2 and Lv-Rescue plasmid. Data were presented as the mean±S.E.M. **C** Gene annotation for enrichment of the candidate genes. **D** Scatter plot results of microarray data presented the differently expressed genes with the cutoff value set at 4/0.25, by comparing the *LINC01225* knockdown group with the control group in MHCC97H and SMCC7721 cells. **E** EGFR mRNA expression level was detected by qRT-PCR. Cells treated with control plasmid, Lv*-*shRNA, Lv-shRNA2 and Lv-Rescue plasmid. Data were presented as the mean±S.E.M. **F** Serum samples were frozen and thawed for five times, and expression stability of *LINC01225* in serum was detected by qRT-PCR. Data were presented as box plots. Box plot explanation: upper horizontal line of box, 75th percentile; lower horizontal line of box, 25th percentile; horizontal bar within box, median; upper horizontal bar outside box, 95th percentile; lower horizontal bar outside box, 5th percentile. All experiments were performed in triplicate. (* *P* < 0.05, ** *P* < 0.01, *** *P* < 0.001)

**Supplementary Figure S2. A** MHCC97H cells were treated with shRNA constructed with Lentivirus packaging plasmid, termed EGFR-shRNA. The effect of EGFR-shRNA on the expression of EGFR in the level of mRNA was detected by qRT-PCR. **B** EGFR protein level of MHCC97H cells treated with EGFR-shRNA was detected by western blot and the IOD value of the bands was evaluated. All experiments were performed in triplicate and presented as the mean±S.E.M. (*** *P* < 0.001).
