# Supplementary figures and images for: LINC01225 promotes occurrence and metastasis of hepatocellular carcinoma in an epidermal growth factor receptor-dependent pathway
Source: Cell Death Dis. 2016 Mar 3;7(3):e2130–. doi: 10.1038/cddis.2016.26 (PMC4823934; doi:10.1038/cddis.2016.26)

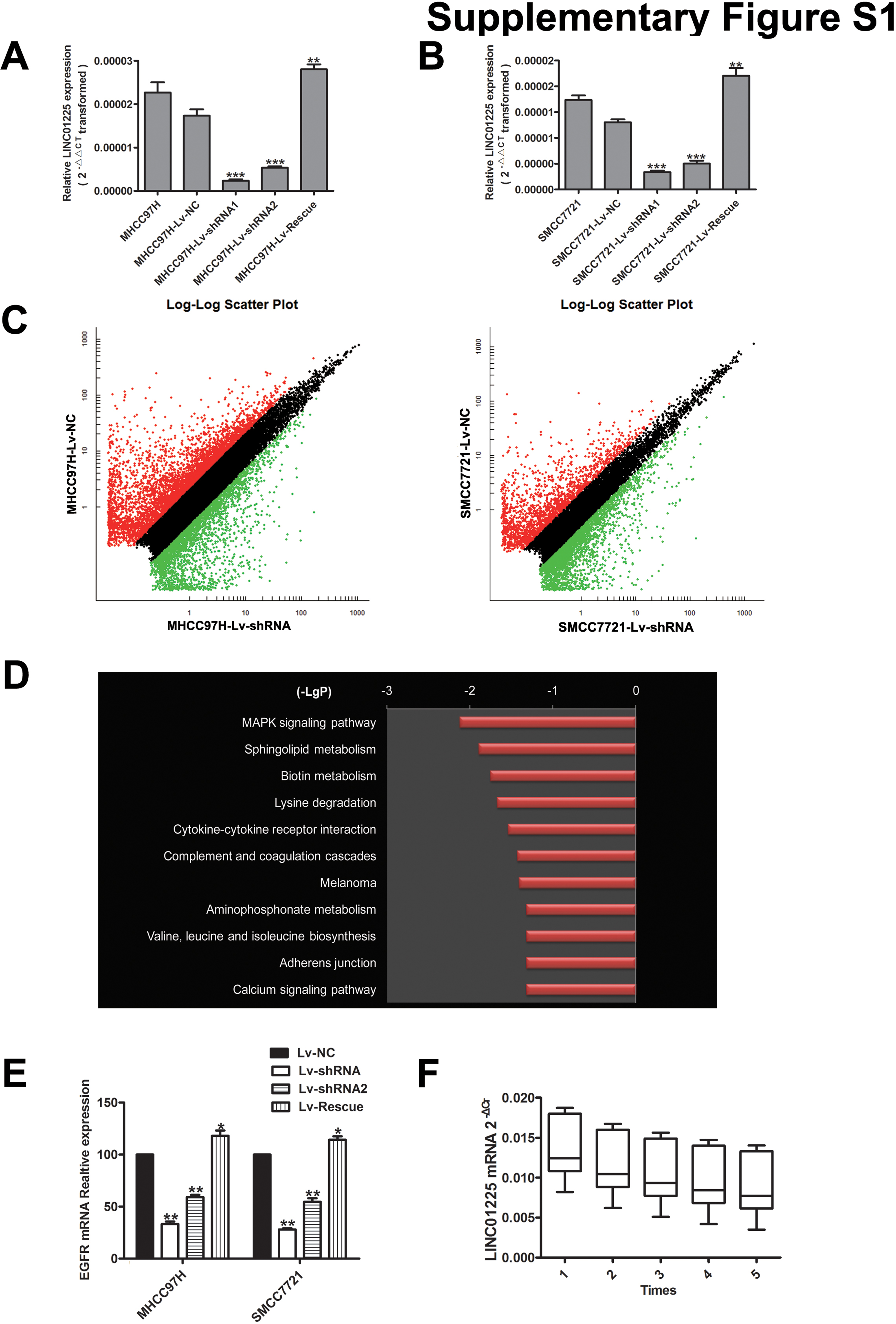

Supplement: Supplementary Figure S1 [file cddis201626x2.tif]

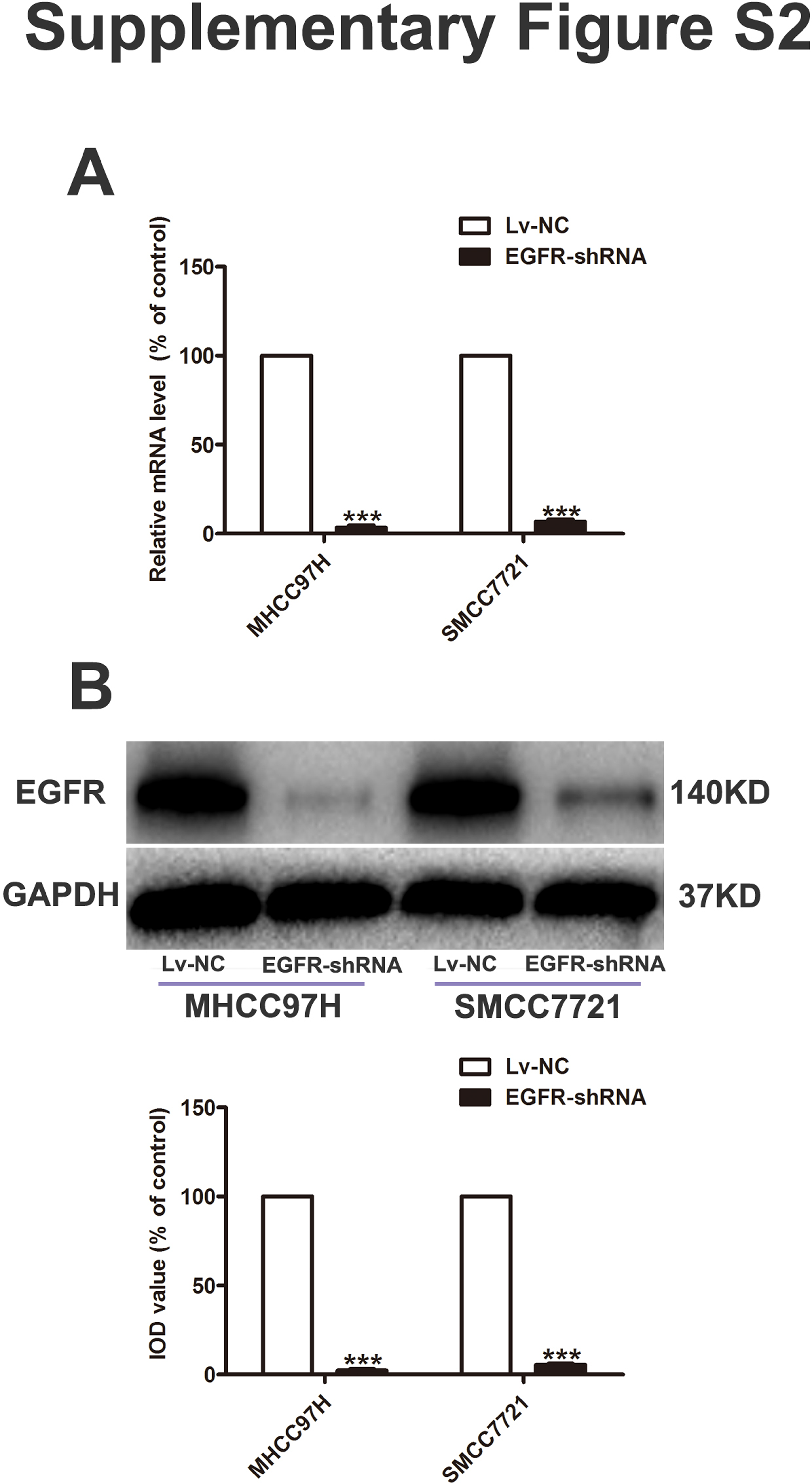

Supplement: Supplementary Figure S2 [file cddis201626x3.tif]
